# Breastfeeding Trends in Cambodia, and the Increased Use of Breast-Milk Substitute—Why Is It a Danger?

**DOI:** 10.3390/nu6072920

**Published:** 2014-07-22

**Authors:** Sophonneary Prak, Miriam Iuell Dahl, Sam Oeurn, Joel Conkle, Aaron Wise, Arnaud Laillou

**Affiliations:** 1 Maternal and Child Health Center, N° 31A, Rue de France (St. 47), 12202 Phnom Penh, Cambodia; E-Mail: sophonprak@gmail.com; 2 UNICEF, Maternal, Newborn Child Health and Nutrition Section, No. 11 Street 75, 12202 Phnom Penh, Cambodia; E-Mails: midahl@unicef.org (M.I.D.); sun@unicef@unicef.org (S.O.); joelconkle@gmail.comorg (J.C.); awise@unicef.org (A.W.)

**Keywords:** breastfeeding, infant and young child feeding, breast-milk substitute, the code of marketing of breast-milk substitutes

## Abstract

A cross-sectional analysis of the Cambodia Demographic Health Surveys from 2000, 2005 and 2010 was conducted to observe the national trends in infant and young child feeding practices. The results showed that rates of exclusive breastfeeding among infants aged 0–5.9 months have increased substantially since 2000, concurrent with an increase in the rates of early initiation of breastfeeding and a reduction in the giving of pre-lacteal feeds. However, the proportion of infants being fed with breast-milk substitutes (BMS) during 0–5.9 months doubled in 5 years (3.4% to 7.0%) from 2000 to 2005, but then did not increase from 2005, likely due to extensive public health campaigns on exclusive breastfeeding. BMS use increased among children aged 6–23.9 months from 2000 to 2010 (4.8% to 9.3%). 26.1% of women delivering in a private clinic provided their child with breast-milk substitute at 0–5.9 months, which is five times more than women delivering in the public sector (5.1%), and the greatest increase in bottle use happened among the urban poor (5.8% to 21.7%). These findings are discussed with reference to the increased supply and marketing of BMS that is occurring in Cambodia.

## 1. Introduction

Cambodia has the 28th highest prevalence of chronic malnutrition in the world, with 40% of children <5 years of age stunted (WAZ < −2 SD), and 28% underweight (WHZ < −2 SD) [1]. As highlighted by many international reports, breastfeeding is one of the most cost effective interventions to improve health and prevent illness in early childhood, however developing countries see a growing trend in the use of breast-milk substitutes (BMS). This is not only a dangerous trend because of the increased risk of morbidity and mortality, but this also greatly undermines the efforts of national policies in regards to achieving the Millennium Development Goals [2,3].

According to the UN Inter-agency Group for Child Mortality Estimation (IGME) 7.6 million children under the age of five die annually in the world [4]. The invisible effects of malnutrition and poor practices of infant and young child feeding (IYCF) inhibit growth and development and increase the susceptibility of disease and death [5]. It is estimated that nutrition-related factors contribute to about 35% of child mortality [6].

The Global Strategy on Infant and Young Child Feeding (GSIYCF) aims to promote, protect and support appropriate feeding practices regardless of the child’s situation, and is the foundation of the current recommendations [7]. Mothers are strongly recommended to initiate breastfeeding within one hour after birth, followed by exclusive breastfeeding (EBF) for six months, with continued breastfeeding for two years or more. At the age of six months the infant should be introduced to safe, age-appropriate foods of solid, semi-solid and soft textures [3].

Breast-milk alone is the ideal nourishment for infants, and contains all the nutrients required for optimal development for the first six months of life. It is safe, easily digested, efficiently used and contains antibodies, in sharp contrast to BMS [3]. The first two years of life is a critical period in regards to growth and development [3]. Energy and nutrient requirements are high and children in this age group are at the greatest risk of nutrient deficiencies [8]. According to Edmond *et al.* [9] 16% of neonatal deaths can be prevented if infants were breastfed from day one, and as much as 22% if breastfeeding started within the first hour after birth. UNICEF [10] states that a non-breastfed child living under unhygienic conditions and in areas with unsafe water is between six to 25 times more likely to die as a result of diarrhea and four times more likely to die of pneumonia than a breastfed child. Breastfeeding is therefore of great importance when it comes to protecting children and decreasing morbidity and mortality rates. A recent analysis from UNICEF and WFP, showed that the economic losses due to sub-optimal breastfeeding is costing Cambodia approximately 24 million USD per year [11].

This study therefore sought to provide information on the trends in infant feeding practices, including the provision of BMS to infants and young children and the use of bottles, among different population subgroups in Cambodia, using nationally collected surveillance data.

## 2. Materials and Methods

### 2.1. Data Sources

UNICEF and the Ministry of Health performed a cross-sectional secondary analysis of the Cambodia Demographic Health Survey (CDHS) [1,12,13] to observe the trends in infant and young child feeding practices such as breastfeeding practices, early initiation of breastfeeding (within one hour after birth), pre-lacteal feeding, the use of BMS, and trends in bottle use. This secondary analysis was based on the three latest CDHS conducted in 2000, 2005 and 2010 and the authors used the most recently available Cambodia DHS datasets from 2000, 2005, to 2010. The CDHS collected data on demographics, socioeconomics and health, and this is a useful and valid source of information on IYCF from a nationally representative sample of households. A total of at least 15,000 households were selected for the survey. The questionnaire was based on model questionnaires developed by the MEASURE DHS project. The three surveys were based on a stratified sample selected in two stages. Stratification was achieved by separating every reporting domain into urban and rural areas. Samples were selected independently in every stratum through a two-stage selection process [1,12,13].

### 2.2. Breastfeeding Indicators

The rate of EBF was defined as the proportion of infants, aged 0–5.9 months, who received only breast-milk and no other fluids or solids except for drops or syrups consisting of vitamins, minerals supplements or medicines [14,15]. The EBF rate was estimated according to the WHO recommended definition of this key IYCF indicator [15] with further categorization for the following age ranges 0–1, 2–3 and 4–5 months of age [15]. It should be noted that EBF does not represent the proportion of infants who are exclusively breastfed from birth until sixth months of age, rather it is a snapshot of current practices for all children 0–5.9 months of age. A wealth index was constructed from data collected in the household questionnaire, and was divided into five categories (from lowest to highest, Q1 to Q5 respectively).

In this study, breast-milk substitute is defined as “any food being marketed or otherwise presented as a partial or total replacement for breast-milk, whether or not suitable for that purpose” [16].

### 2.3. Statistical Analysis

Before conducting statistical tests to understand the changes within groups over time, the basic prevalence of all indicators were calculated for each subgroup and survey (*i.e.*, 2000, 2005, and 2010). Then linear regressions were run for breastfeeding practices (exclusive breastfeeding, early initiation, pre-lacteal feeding, and breast-milk substitute and bottle use) to determine whether, for each subgroup, the absolute prevalence differences observed over each timeframe were statistically significant. The significance of these differences was estimated using: *Y* = α*_i_* + δ *T* + ε (*Y*: indicator difference; *T*: binary timeframe variable; α: constant term (prevalence of timeframe variable for subgroup *i*); δ: change in by timeframe and; ε: error term).

Finally, linear regressions were run to determine whether the absolute prevalence differences observed within equity groups over each timeframe were statistically significant. The significance of this difference of differences was estimated using: *Y* = α_i,*h*_ + β *E* + δ *T* + γ *E*•*T* + ε (*Y*: change; *E*: binary subgroup variable; *T*: binary timeframe variable; E•T: interaction term (product of subgroup and timeframe variables); α: constant term (prevalence of subgroup *i* for timeframe *h*); β: change in indicator by subgroup; δ: change in indicator by timeframe; γ: Difference in changes in indicator by subgroup and timeframe; and ε: error term).

All of the analyses were carried out using Stata MP (version 11, StataCorp LP, College Station, TX, USA). The complex sampling design of DHS surveys was accounted for throughout the project using Stata’s svyset function. Variance estimations with one or more missing PSUs were centered. Sub-population variables were created for every linear regression in accordance with the unconditional approach.

## 3. Results

**Figure 1:**
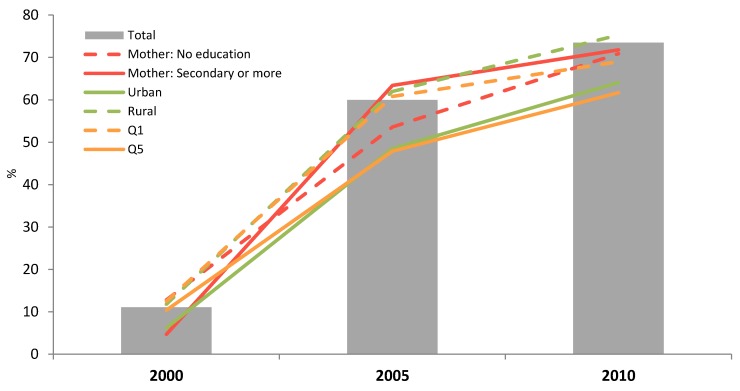
Exclusive breastfeeding (0–5.9 months) by year and according to the mother’s education, household wealth (richest = Q5; poorest = Q1) or living area (*n* = 801, 741 and 704 in 2000, 2005 and 2010 respectively).

The secondary analysis of the data on breastfeeding trends (Figure 1) from the latest CDHS showed that EBF (0–5.9 months) significantly increased from 11.1% in 2000 to 73.5% in 2010 (*p* < 0.05). While the greatest significant improvement was seen among the mothers with secondary school education or higher between 2000 and 2005 (4.7% to 63.4%, *p* < 0.05), there was improvement among all levels of education and by 2005 there was no significant difference in EBF by level of education. In 2005 the only significant subgroup difference was that children in urban areas were less likely to be exclusively breastfed (48.5% *vs.* 62.0%, *p* < 05). From 2005 to 2010 significant improvements (*p* < 0.05) were observed for each subgroup, except for mothers with secondary school education or higher and the lowest and highest wealth quintiles, quintile 1 (Q1) and quintile 5 (Q5) respectively. Over the same time period the gap between urban and rural areas narrowed slightly and was no longer statistically significant (64.1 *vs.* 75.3). By 2010 there were no significant differences within the subgroups of sex, education, residence or wealth. Between 2000 and 2010, there was equitable improvement in EBF as equity gaps narrowed and the rate of change between subgroups was the same (*p* > 0.05).

In the same period, the findings indicate that early initiation of breastfeeding has significantly increased over the years while pre-lacteal feeding (Feeding of a newborn baby with carbohydrate-electrolyte solutions) has decreased from 2000 to 2010, with 65.8% of the children being breastfed within the first hour after birth in 2010, compared to only 11.1% in 2000 (*p* < 0.05). However, women delivering in private facilities were two times more likely to give a pre-lacteal feed compared to women delivering in public facilities (34.1% *versus* 15.2%, *p* < 0.05) (Table 1).

**Table 1:** Early initiation of breastfeeding and pre-lacteal feeding by place of delivery, Cambodia Demographic Health Survey (CDHS) 2000–2010 *.

Facility	Early Initiation (<2 Years)			Prelacteal (<2 Years)		
	2000	2005	2010	2000	2005	2010
**Public**	17.4 ^a^	45.3 ^a^	69.9 ^b^	92.8	32.8 ^b^	15.2 ^b^
**Private**	13.0	35.6	66.0	90.5	52.6	34.1
**Home**	10.5	32.9	59.3	94.1	47.0	21.4

Note: * 2000 data recalculated to include water; ^a^ significantly different from Home; and ^b^ significantly different from Private and Home (*p* < 0.05).

**Figure 2:**
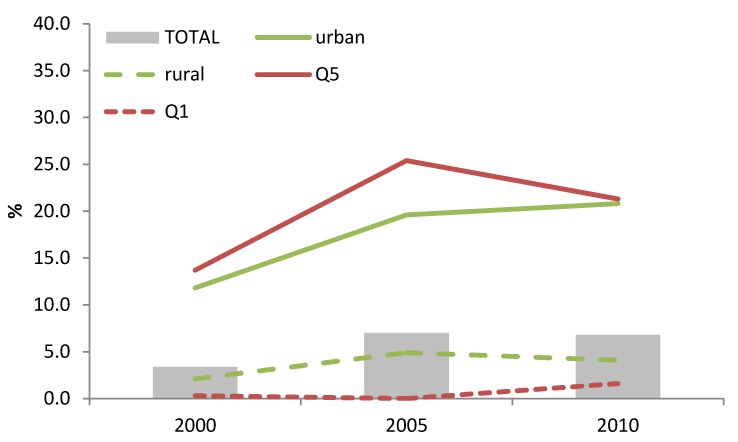
Trends in breast-milk substitutes (BMS) use among children 0–5.9 months of age according to maternal education, household wealth (richest = Q5; poorest = Q1) or living area.

There was a significant increase in the use of BMS among infants aged 0–5.9 months from 3.4% in 2000 to 7.0% in 2005 (*p* < 0.05), but from 2005 to 2010 there was no further increase in BMS use. However, approximately 21% of infants (0–5.9 months) from the urban region and in the wealthiest quintile were fed with BMS in 2010 (Figure 2). In addition, 26.1% of the women delivering in a private clinic provided their child with BMS in 2010, which was five times more than women delivering in the public sector (5.1%) and therefore significantly different (*p* < 0.05).

**Figure 3:**
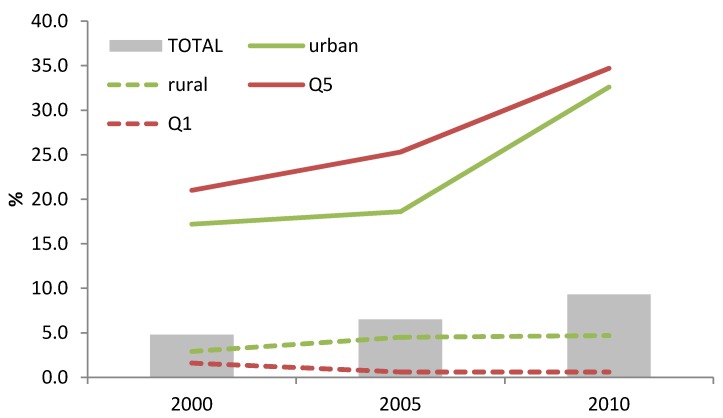
Trends in BMS use among children 6–23.9 months of age according to maternal education, household wealth (richest = Q5; poorest = Q1) or living area.

Among children aged 6–23.9 months, the use of BMS has increased since 2000. Among the wealthiest quintile (Q5), the consumption rose from 25.3% to 34.7% between 2005 and 2010 (*p* < 0.05). One third of the children (6–23.9 months) from the urban quintile were fed with BMS (Figure 3). Children born in a private facility were found to be more at risk of being fed with BMS (26.9% *versus* 10.1%, in private and public facilities respectively, in 2010; *p* < 0.05). Overall, the consumption of BMS among children aged 6–23.9 months almost doubled in 10 years (4.8% to 9.3% from 2000 to 2010; *p* < 0.05).

**Figure 4:**
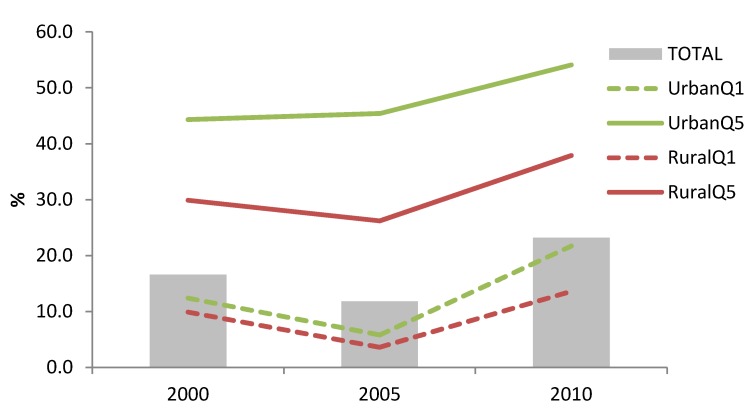
Trends in bottle use among children 0–23.9 months of age according to maternal education, household wealth (richest = Q5; poorest = Q1) or living area to nurse an infant with liquid food from a nursing bottle.

In addition to BMS use, there was an increase in bottle use among children aged 0–23.9 months in both urban and rural areas from 2005 to 2010 (30.1% to 45.3%, and 8.7% to 18.9%, respectively; *p* < 0.05), as well as among the highest and poorest wealth quintiles (35.4% to 48.5%, and 3.7% to 13.7%, respectively; *p* < 0.05). The greatest increase in bottle use has happened among urban poor (5.8% to 21.7%, *p* < 0.05) (Figure 4).

Nutrition counseling through antenatal care (ANC) was higher in urban areas compared to rural areas (90.9% and 79.5%, respectively), and was higher among the mothers with secondary school education (76.1% for women with no education *versus* 88.8% for secondary education or higher) and the wealthier mothers (76.8% for the poorest quintile *versus* 89.7% for the wealthiest quintile).

## 4. Discussion

Cambodia has made great improvements since 2000 in regards to early initiation of breastfeeding, EBF rates and pre-lacteal feeding. One reason for this might be public health campaigns focusing on appropriate IYCF, as they have been shown to be an effective intervention in regards to promoting and protecting breastfeeding.

One of the major findings from this research is that the use of BMS has not increased among children aged 0–5.9 months after 2005. This is likely a result of a nationwide communication campaign on exclusive breastfeeding that utilized mass media and interpersonal communication. This campaign did not just have an effect on BMS use, but also on the provision of water to newborns and early introduction of complementary foods. However, there are limited resources to continue a communication campaign indefinitely, and when the campaign stops, illegal promotion of BMS may cause an increase in use among this vulnerable age group.

In regards to children aged 6–23.9 months, the situation is different. After modest increases in the use of BMS for the urban and wealthy population from 2000 to 2005, there was a sharp increase in BMS use after 2005. For urban areas, the use of BMS among children 6–23.9 months nearly doubled from 2005 to 2010. For rural areas and the poorest wealth quintile, there was no change over the last five years. These trends may be the result of increased promotion of “follow-on milk” as highlighted further down in the discussion.

The findings indicate that early initiation of breastfeeding has increased while pre-lacteal feeding has decreased since 2000. The two indicators have a causal relationship, and pre-lacteal feeding was the major reason for not initiating breastfeeding early [17]. Table 1 showed that women delivering in a public or private facility were more likely to initiate breastfeeding early when compared to the women delivering at home. There was only a slight difference between public and private facilities, which might suggest that both facilities are promoting early initiation of breastfeeding. However, women delivering in private facilities were shown to be over two times more likely to give a pre-lacteal feed when compared to women delivering in a public facility or at home. This might be a result of the private sector promoting the use of BMS.

Additionally, private health facilities seems to less advocate for exclusive breastfeeding until the age of 6 months as 1 in 4 of the women delivering in a private clinic use BMS, which is three times more than women delivering in the public sector. This might be explained by women delivering in private facilities are wealthier and more able to purchase BMS, as well as the possibility that the wealthier women might have jobs, and need to return to work. However, this difference might also be explained by the illegal promotion of BMS by private health workers or any other private professional with access to the private clinic, and the increased access to large amounts and multiple brands of BMS.

The results show that EBF and BMS use has increased over the last years. The low rate of both EBF and BMS use in 2000 can be explained by provision of other liquids and early initiation of complementary feeding, where 70.1% of children less than two months of age were given water, 4.4% were given non-milk liquids, such as juice or tea, and 4.1% were given solid or semi-solid foods [13]. According to the latest CDHS [1] the provision of water has decreased substantially to only 3.8% in 2010, along with 0% receiving non-milk liquids and 0.7% receiving complementary foods. These findings imply that Cambodia has seen great improvements in regards to the provision of other liquids and foods over the last years but not BMS.

Even though the levels of early initiation and EBF have increased substantially in Cambodia, the illegal promotion of breast-milk substitutes [2] by the private sector and others could be threatening the recent gains made in IYCF. These are important issues that need to be addressed, and this further stresses the importance of enforcing compliance of the International Code of Marketing of Breast-milk Substitutes.

There is a need to examine hospital practices and ensure that breastfeeding and appropriate complementary feeding is being adequately promoted in private facilities and that BMS is not given unless absolutely necessary. However, it seems more likely that the increased use of BMS is related to wealth and access to these products, and cannot only be blamed on the aggressive marketing from the manufacturers.

Breast-milk is well known to be both economically and physiologically essential to child survival, nevertheless the marketing of breast-milk substitutes and the increased availability of BMS has dislodged breastfeeding as a feasible and desirable strategy for infant feeding [18]. In our study, 1/5th of the urban infants (0–5.9 months) were being fed with BMS. A recent press release [19] dated on the 7th of May 2014 from UNICEF, WHO and 3 other NGOs highlighted that there are 113 different breast-milk substitutes available on the market in Phnom Penh alone, and so far we have not found any that adhere entirely to the government’s legislation, the Sub-decree 133 [20], which follows the International Code of Marketing of Breast-milk Substitutes [16]. A local journal also highlighted that 41 percent of stores selling BMS in Phnom Penh had in-store promotions for the products [21], which is banned by Sub-Decree 133. This sub-decree on marketing of products for IYCF were approved in 2005 by the Cambodian government. The aim was to support good nutrition for infants and children, as well as support, protect and promote optimal breastfeeding practices, including early initiation of breastfeeding, EBF until 6 months of age and continued breastfeeding for two years and beyond by banning promotion of BMS.

As emphasized previously, a variety of different BMS products are becoming increasingly available in low income countries. An additional concern in Cambodia is the vast amount of imported brands that do not provide instructions or information written in Khmer. This, in addition to misleading pictures and messages to idealize formula, may be contributing to low levels of understanding causing inappropriate use of BMS [22]. Mothers are attracted to the new products on display and in-store promotions, however they may not be able to differentiate between appropriate and inappropriate products [2]. An example of this is found in the research conducted in Laos by Barennes *et al.* [23], where they found a misleading impact of a label on a coffee creamer which showed a bear holding its cub in the breastfeeding position. This caused 18% of the study population giving this creamer to their infants, believing it was a suitable BMS, resulting in several cases of severe malnutrition and deaths. Despite the obvious harm caused to infants worldwide by the BMS industry’s methods, their misleading advertising continues, largely because compliance with the International Code of Marketing of Breast-milk Substitutes is not enforced [24].

Our results show a substantial increase in bottle feeding (0–23.9 months) between 2000 and 2010, especially among children older than six months, however, as BMS use did not increase among all groups, the increase in bottle use cannot only be related to BMS use. According to a report from IBFAN bottles are also being used for juice, water, sugar water, diluted condensed milk, and rice porridge [25], indicating that public education around the provision of these fluids and the use of bottles may be needed. Due to the public health campaigns on the importance of EBF until the age of 6 months, less bottle feeding is seen in this age group. However, the greatest increase in bottle use was found in rural areas and among urban poor (from 5.8% in 2005 to 21.7% in 2010). This is of particular concern because of poor sanitation in these areas, and indicates the need for messages concerning the health and contamination risk that bottles and teats pose.

Antenatal care (ANC) counseling, where breastfeeding messages should be provided, seems to be delivered to a higher proportion of the urban and/or the wealthiest quintile. However, the pregnant women receiving the most counseling exhibit the poorest breastfeeding practices, suggesting that the content of the counseling needs to be examined to ensure that breastfeeding is being adequately promoted as the optimal method of infant feeding. Our finding does not support previous research conducted by Bhandari *et al.* [26], who found that the mothers’ level of education affected their feeding behavior, and showed that educational interventions improved feeding practices.

## 5. Conclusions

Cambodia has made great improvements in regards to early initiation of breastfeeding, exclusive breastfeeding rates and pre-lacteal feeding, however the use of breast-milk substitutes and bottle feeding has increased among children >6 months. Unsafe bottle feeding such as dilution or preparation with unsanitary water puts the child at risk of infectious diseases, diarrhea and malnutrition, increasing the chance of mortality. In order to improve the health of Cambodia’s children there is a need for promotion, protection and support of optimal breastfeeding and complementary feeding practices. The efforts to improve feeding practices should be based on the assessment of existing practices at community and health facility levels, and the identification of bottlenecks, such as maternity leave, is essential. More research is needed on the marketing and increased access of breast-milk substitutes in Cambodia, and South East Asia as a whole.
